# Optimization of a Spin-Orbit Torque Switching Scheme Based on Micromagnetic Simulations and Reinforcement Learning

**DOI:** 10.3390/mi12040443

**Published:** 2021-04-15

**Authors:** Roberto L. de Orio, Johannes Ender, Simone Fiorentini, Wolfgang Goes, Siegfried Selberherr, Viktor Sverdlov

**Affiliations:** 1 Institute for Microelectronics, TU Wien, Gußhausstraße 27-29/E360, 1040 Vienna, Austria; Selberherr@TUWien.ac.at; 2 Christian Doppler Laboratory for Nonvolatile Magnetoresistive Memory and Logic at the Institute for Microelectronics, TU Wien, 1040 Vienna, Austria; ender@iue.tuwien.ac.at (J.E.); fiorentini@iue.tuwien.ac.at (S.F.); sverdlov@iue.tuwien.ac.at (V.S.); 3 Silvaco Europe Ltd., Cambridge PE27 5JL, UK; wolfgang.goes@silvaco.com

**Keywords:** spin-orbit torque MRAM, reinforcement learning, two-pulse switching scheme, magnetic field-free switching, machine learning

## Abstract

Spin-orbit torque memory is a suitable candidate for next generation nonvolatile magnetoresistive random access memory. It combines high-speed operation with excellent endurance, being particularly promising for application in caches. In this work, a two-current pulse magnetic field-free spin-orbit torque switching scheme is combined with reinforcement learning in order to determine current pulse parameters leading to the fastest magnetization switching for the scheme. Based on micromagnetic simulations, it is shown that the switching probability strongly depends on the configuration of the current pulses for cell operation with sub-nanosecond timing. We demonstrate that the implemented reinforcement learning setup is able to determine an optimal pulse configuration to achieve a switching time in the order of 150 ps, which is 50% shorter than the time obtained with non-optimized pulse parameters. Reinforcement learning is a promising tool to automate and further optimize the switching characteristics of the two-pulse scheme. An analysis of the impact of material parameter variations has shown that deterministic switching can be ensured for all cells within the variation space, provided that the current densities of the applied pulses are properly adjusted.

## 1. Introduction

Spin-transfer torque magnetoresistive random access memory (STT-MRAM) is currently the state-of-the-art MRAM technology, entering volume production at all major foundries [1,2,3,4,5,6]. It is an emerging nonvolatile technology suitable for future universal memory applications. One of its key advantages is that it is compatible with CMOS technology, so it can be straightforwardly embedded in circuits [7]. It is promising not only for standalone, but also for embedded memory applications as replacement of conventional volatile CMOS-based and nonvolatile flash memories in systems on chip. STT-MRAM can be integrated in a broad range of applications, from Internet-of-Things to automotive applications [3] and last level caches [8,9,10]. Recently, 1Gb standalone [11] and embedded STT-MRAM solutions [2,4,12,13] have been reported and STT-MRAM operation with a timing of a few nanoseconds has been demonstrated [8]. However, in order to further reduce the timing below the nanosecond range, the required current density becomes quite large. This creates an important limitation, since large currents flowing through the thin tunnel oxide of a magnetic tunnel junction (MTJ) lead to reliability issues, reducing the MRAM endurance.

Spin-orbit torque (SOT) MRAM is a promising nonvolatile memory candidate outperforming STT-MRAM for ultra-fast operation [14]. In SOT-MRAM, the large current required for the writing operation does not flow through the MTJ. Switching is accomplished by applying a current through a heavy metal wire attached to the magnetic free layer (FL). Thus, it can operate with a sub-nanosecond timing retaining excellent endurance [15,16,17]. These properties make SOT-MRAM particularly interesting for nonvolatile replacement of the classical static random access memory (SRAM) used in caches. It should be pointed out, however, that deterministic SOT switching of a perpendicularly magnetized FL requires an external magnetic field [18]. Several field-free schemes have been proposed to circumvent this issue, usually at the cost of a more complex cell stack fabrication [15,16,19,20,21,22,23].

We consider an alternative field-free scheme, in which a purely electrical control of the switching process is realized by applying current pulses to two orthogonal heavy metal wires [24]. A proper configuration of the current pulses is able to reverse the perpendicularly magnetized FL [25]. Nevertheless, an interesting question arises: how can one determine a pulse sequence that leads to optimal switching? Searching for such a pulse sequence requires a very large number of experiments and/or simulations. Ideally, this task can be outsourced to an algorithm and performed in a guided and automated way.

Machine learning (ML) has been increasingly applied to the solution of physics-based problems [26] and has already been used to solve fundamental micromagnetic problems, such as the computation of the magnetization dynamics of a thin film [27] and of the magnetic microstructure of a single magnetic body [28]. Recently, an ML model was applied to identify the regime of field-free SOT switching as a function of the magnitude of the applied current density, the nanomagnet size, and the interfacial Dyzaloshinskii-Moriya interaction [29]. The two most used ML approaches are supervised and unsupervised learning. In turn, another sub-branch of ML so-called reinforcement learning (RL) has gained interest [30,31]. Big RL breakthroughs were achieved lately using games like chess or Go [32], but this type of learning algorithm has also been successfully applied for physics-based problems.

The RL principle of operation is based on an agent and an environment, in such a way that the agent interacts with the environment and learns how to act or take decisions to achieve a specific state or goal [30]. In other words, the agent interacts with the environment by performing actions that cause the environment to move from one state to another. Once the environment moves to a new state, it informs about its state and returns a reward to the agent. Based on this information, the agent can decide to take actions to maximize the cumulative reward received over time. During this process, the agent learns how to achieve the given objective.

In this work, we combine the two-current pulse switching scheme with an RL algorithm to optimize the switching of a spin-orbit torque memory cell. We demonstrate that the reinforcement learning implementation can find an optimal sequence and timing for the current pulses in order to achieve faster switching in comparison to a conventional combination of pulse parameters.

## 2. Spin-Orbit Torque Memory Cell and Switching Dynamics

**Figure 1:**
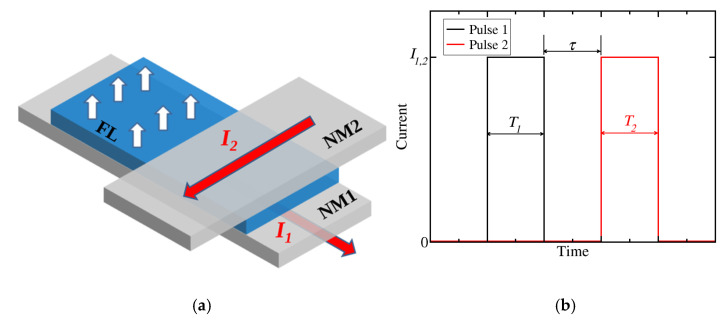
(**a**) SOT-MRAM cell for switching based on (**b**) two orthogonal current pulses. Pulse 1 is applied to the NM1 wire and Pulse 2 is applied to the NM2 wire. *I_1,2_* is the current amplitude and *T_1,2_* is the width of the first/second pulse. *τ* represents the delay or overlap between the pulses.

The two-pulse switching scheme for a SOT memory cell is depicted in Figure 1. The cell is formed by growing a perpendicularly magnetized FL on top of a heavy metal wire (NM1), where a first current pulse is applied to generate the initial SOT on the FL. On the right part of the cell, a second, orthogonal heavy metal wire (NM2) is placed on top of the FL, and a second current pulse is applied through it. The SOT generated due to this second pulse acts on the FL to complete the magnetization switching of the memory cell [33]. The NM1/FL/NM2 stack composes the structural part used for the writing operation of the memory cell. In the left part of the cell, next to the SOT writing stack, an MTJ is grown on top of the FL, which is required for carrying out the reading operation of the memory cell via measurement of the tunneling magnetoresistance.

In order to carry out micromagnetic simulations of the two-pulse SOT switching scheme, the magnetization dynamics is described by the Landau-Lifshitz-Gilbert (LLG) equation

(1) $$\frac{\partial m}{\partial t}=-\gamma \mu_{0\;}m\times H_{eff}+\alpha m\times \frac{\partial m}{\partial t}\;-\gamma \frac{\hbar }{2e}\frac{\theta_{SH}j_{1}}{M_{S}d}\left[m\times \left(m\times y\right)\right]\Theta \left(t,T_{1}\right)\;+\gamma \frac{\hbar }{2e}\frac{\theta_{SH}j_{2}}{M_{S}d}\left[m\times \left(m\times x\right)\right]\Theta \left(t,T_{1},T_{2}\right)$$

where **m** is the normalized magnetization, *γ* is the gyromagnetic ratio, *µ*_0_ is the vacuum permeability, *α* is the Gilbert damping factor, and *M_S_* is the saturation magnetization. **H_eff_** is an effective magnetic field, which includes the exchange field, the uniaxial perpendicular anisotropy field, the demagnetization field, the current-induced field, and the stochastic thermal field at 300 K. The last two terms on the right-hand side of the LLG equation describe the SOT generated by the applied current pulses through the NM1 and the NM2 wire, respectively, where *e* is the elementary charge, *ħ* is the reduced Plank constant, *θ_SH_* is an effective Hall angle, *j_1,2_* is the current density of the first/second pulse, *d* is the FL thickness, and Θ(∙) is a function which determines when each pulse is active.

Equation (1) is solved numerically using a micromagnetic simulation software developed in-house [34] based on the finite difference method. The simulation parameters are given in Table 1.

## 3. Reinforcement Learning for the Two-Pulse Spin-Orbit Torque Switching

**Figure 2:**
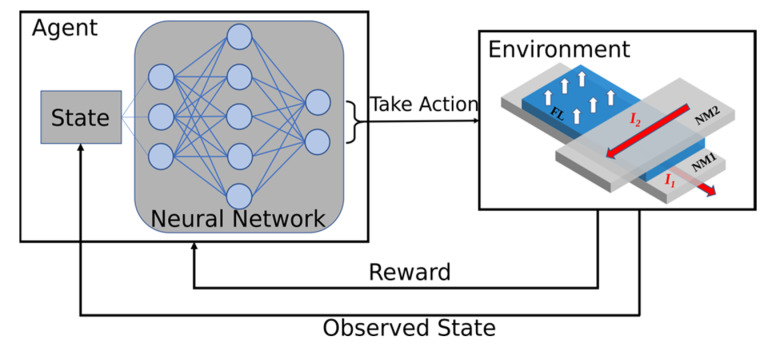
Reinforcement learning setup for the two-pulse switching scheme. The micromagnetic simulation of the memory cell provides the environment, with which the agent interacts and takes actions to achieve the fastest magnetization switching.

Figure 2 shows the RL setup implemented for performing the learning experiments with the two-pulse switching scheme. The environment consists of our in-house tool, which provides the simulation of the memory cell switching and returns the current state of the simulation together with a reward after every iteration. The used deep Q-network (DQN) algorithm [31] incorporates a neural network to approximate a function for mapping states to actions. An existing Python library providing the RL capabilities has been employed [35]. Here, the goal of our RL implementation is to determine the pulse configuration which results in the shortest switching time, defined as the time when the perpendicular component of the magnetization vector reaches −0.5, i.e., *m_z_* = −0.5.

The state vector returned from the environment after every iteration consists of 11 variables: the average of the three magnetization vector components (*m_x_*, *m_y_*, *m_z_*), the difference of each component to the previous iteration (*Δm_x_*, *Δm_y_*, *Δm_z_*), the average component of the effective magnetic field (*H_eff,x_*, *H_eff,y_*, *H_eff,z_*), and two variables indicating whether the first and the second pulse are active or not. Based on the state information, the learning agent deduces which action to take. It is important that the dynamics of the magnetization vector, given by (*Δm_x_*, *Δm_y_*, *Δm_z_*), is taken into account, so the direction in which the magnetization is moving is known. In this way, the agent can decide on the best action to take to drive the switching as fast as possible. Our setup allows the agent to take four different actions, namely, setting both pulses off, setting both pulses on, turn the first pulse on with the second off, or turn the first pulse off and the second pulse on. If a pulse is on, it means that current has been applied to the corresponding heavy metal wire and a spin torque is applied to the magnetization of the FL.

The rewarding scheme is critical for the RL approach, because it is the main factor which leads the learning algorithm in the right direction and the agent to select the best actions to achieve the target. The reward is an integer value returned by the environment, indicating whether the actions performed by the agent were good or bad. For the SOT switching, the rewarding scheme is chosen such that a shorter switching time corresponds to a higher reward, since the RL algorithm tries to maximize the cumulative reward during the learning process. Here, a reward of −1 is given for every simulation step in which the target, *m_z_* = −0.5, has not been reached yet. We define *t_max_* = 1 ns as an upper limit for the simulation time. If the target is not reached within this time, the learning episode is terminated and a new one is started. On the other hand, if the target is reached at a time *t_final_* before *t_max_*, a positive reward of (*t_max_*−*t_final_*)/*Δt* is returned, where *Δt* is the simulation time-step. In this way, the rewarding scheme is a complementary measure of the number of time-steps required to reach the switching. The smaller the number of time-steps needed to switch, the shorter the switching time is and, therefore, the larger the reward is.

## 4. Results and Discussion

### 4.1. Numerical Simulations

Micromagnetic simulations of the switching dynamics of the two-pulse SOT scheme, as described in Section 2, were carried out. We start by investigating the impact of the pulse configuration on the magnetization dynamics. In particular, the current densities of the first and the second current pulse are fixed at *j*_1_ = 2.7 × 10^12^ A/m^2^ and *j*_2_ = 1.3 × 10^12^ A/m^2^, respectively, while the pulse durations *T*_1_ and *T*_2_ can be modified (c.f. Figure 1). A perfect synchronization between the pulses is considered, i.e., the second current pulse is turned on immediately after the first pulse is turned off. Thus, there is no delay or overlap between the pulses (*τ* = 0). This constraint will be lifted in Section 4.2, where the results of the RL approach are discussed.

**Figure 3:**
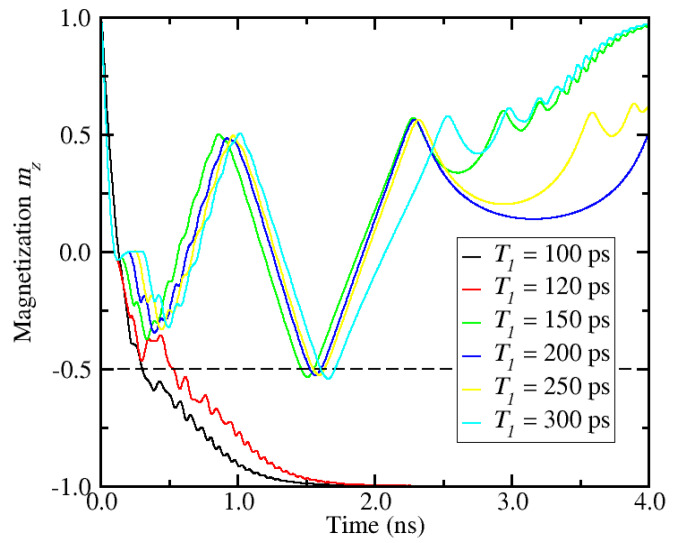
Perpendicular component of the magnetization vector (average of 50 realizations) as a function of time for various durations of the first pulse, *T*_1_. The simulation parameters are found in Table 1 and *j*_1_ = 2.7 × 10^12^ A/m^2^, *j*_2_ = 1.3 × 10^12^ A/m^2^, and *T*_2_ = 100 ps. *j*_1_ and *T*_1_ are the current density and the duration of the first pulse, respectively, and *j*_2_ and *T*_2_ are the current density and the duration of the second pulse (c.f. Figure 1b). The dashed line represents the switching threshold.

Figure 3 shows the perpendicular component of the magnetization (*m_z_*) as a function of time for different widths of the first current pulse, while the second pulse width is kept fixed at *T*_2_ = 100 ps. In order to account for the thermal spread resulting from the stochastic thermal field at room temperature, a total of 50 realizations are considered for each simulation condition. The curves shown in Figure 3 represent the average of these 50 realizations. One can clearly see that, depending on the width of the first pulse, the magnetization dynamics changes significantly, and so does the switching behavior. Here, the pulse sequence and the timing lead to successful magnetization reversal, when the width of the first pulse is short, while switching does not occur for larger values of *T*_1_.

**Figure 4:**
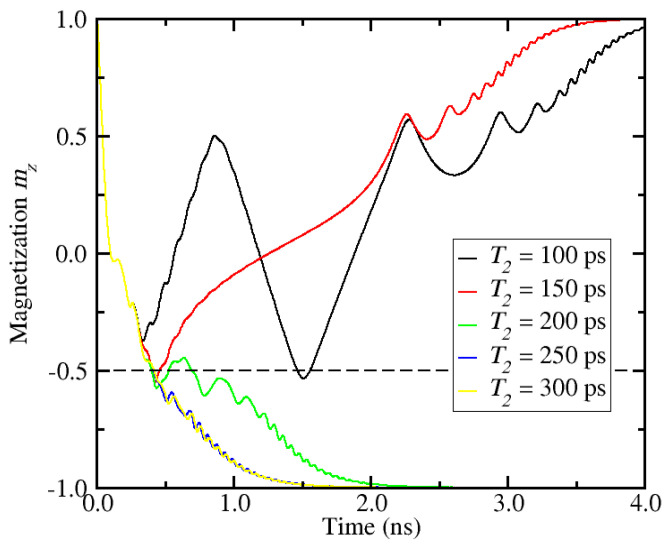
Switching dynamics for different values of the second pulse duration *T*_2_ for a fixed first pulse width *T*_1_ = 150 ps. The dashed line represents the switching threshold.

Next, we reverse the analysis and fix the first current pulse width at *T*_1_ = 150 ps, while the width of the second current pulse is varied. The resulting magnetization dynamics is shown in Figure 4. As in the previous results, switching is obtained depending on the value of *T*_2_. In contrast to the previous scenario, successful switching is observed as the second pulse width becomes longer.

**Figure 5:**
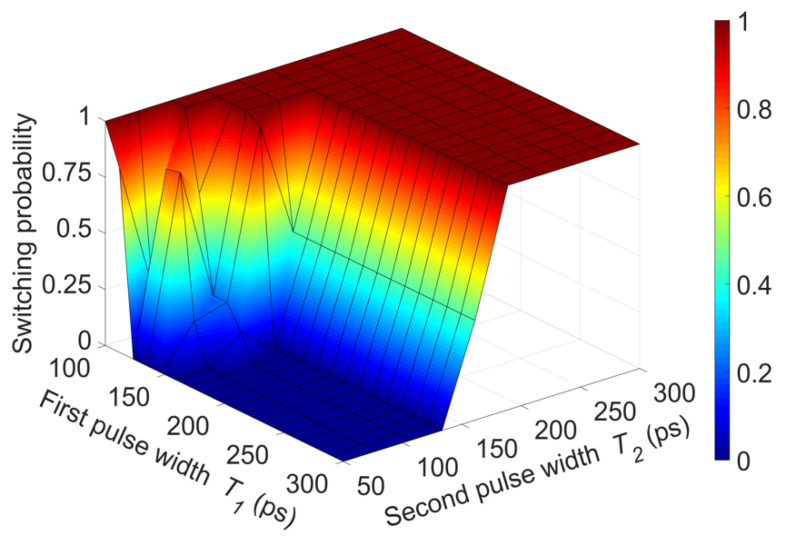
Switching probability as a function of the first and the second current pulse widths, *T*_1_, *T*_2_. For short pulse widths, precise pulse schemes are required to obtain deterministic switching.

The above results suggest that the configuration of the pulse sequence has an important impact on the switching characteristics of the cell, in such a way that variations of the pulse configuration can lead to either switching or non-switching schemes. To further understand this impact, we performed simulations for various combinations of pulses and evaluate the switching probability. The results are shown in Figure 5, which plots the switching probability as a function of the first and the second pulse width. In general, for short values of *T*_2_ (≤150 ps), the switching probability depends largely on the first pulse width, i.e., it depends on the particular pulse sequence and small changes of the pulses can yield successful or non-successful magnetization switching. In turn, increasing *T*_2_ beyond ~200 ps, the switching probability tends to 1, becoming practically insensitive to the duration of the first pulse.

From the previous analysis, we are able to determine pulse parameters that lead to deterministic switching of the memory cell. However, this does not guarantee that these parameters produce fast switching. Now we would like to find the pulse sequence which leads to the fastest possible switching. In order to accomplish that, we have to evaluate many more combinations of pulse sequences than those considered before. It should be pointed out that the previous results were obtained by manually running a total of 180 micromagnetic simulations. Considering that 50 realizations (due to the stochastic thermal field) are carried out for each pulse sequence combination, the number of switching simulations increases to 9000, even though delays or overlaps between the pulses are still not considered. Thus, taking into account all possible variations of pulse parameters results in an exponential increase of the required number of simulations, which makes a manual optimization of the switching intractable. Here, the RL setup described in Section 3 is extremely useful, offering a powerful methodology for searching the fastest switching condition in a guided way.

### 4.2. Reinforcement Learning Experiments

RL is applied with the goal of achieving the fastest magnetization switching, namely to achieve the shortest switching time, which is determined by the time when the condition *m_z_* = −0.5 is reached. The agent searches for a pulse sequence and combination of the first and the second pulse duration, *T*_1_, *T*_2_, which lead to the shortest switching time. The actions performed by the agent (c.f. Figure 2) have been restricted to facilitate the learning process, thus it can switch on and off each pulse individually. However, the pulse synchronization constraint of the previous section is now relaxed, so that the current pulses are allowed to overlap or be delayed. The minimum pulse width is limited to 100 ps and the amplitude of the pulse is fixed to 130 µA and 100 µA for the first and the second current pulse, respectively. A learning episode is finished once *m_z_* = −0.5 or the time has reached 1 ns.

**Figure 6:**
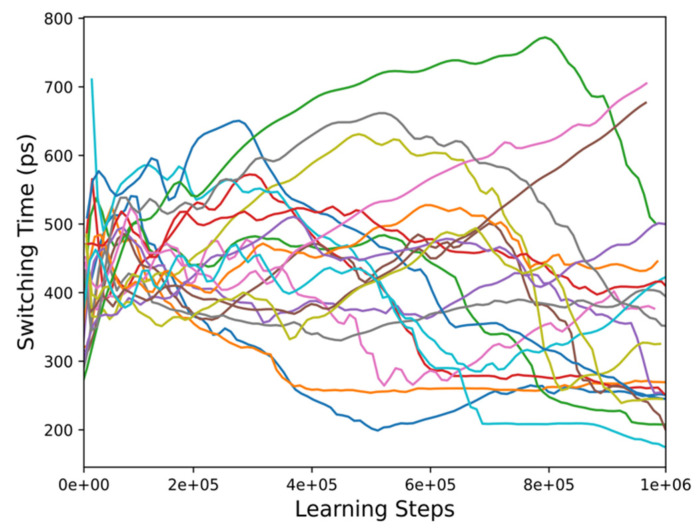
Switching time over the course of the learning period of 20 independent runs. As the number of learning steps increases, there is a trend towards switching time reduction.

**Figure 7:**
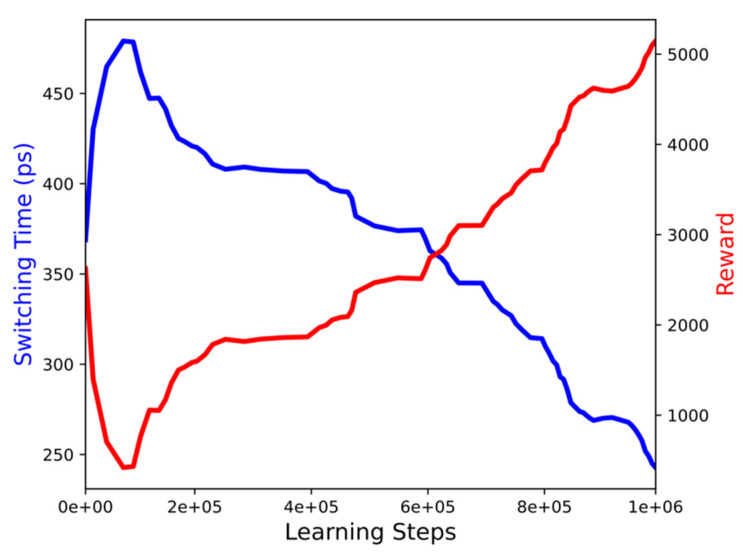
Learning curve showing the mean switching time and reward over 10^6^ time steps. It shows that, the faster the cell switches, the larger is the accumulated reward during learning.

The results of the learning process of our RL setting are shown in Figure 6 and Figure 7, respectively. Figure 6 reports the switching time over the course of the learning period for 20 independent learning runs, where each run encompasses 10^6^ learning steps. During an initial exploration phase, the action selection by the agent is not greedy, i.e., an action is not selected with the purpose of accumulating the highest reward, but the agent takes a random action to explore the state-action space. Furthermore, different random seeds are used for initializing the neural network weights. A general trend can, however, be observed, which is the reduction of the switching time as the number of learning steps increases. Initially, the switching times are distributed around 400–500 ps, but as the number of learning steps increases, several runs reach switching times in the 200–300 ps range.

The switching time decrease with the learning progress can be better visualized in Figure 7, which shows the mean switching time and the reward as a function of the number of learning steps of the six best learning runs. First, an increase of the switching time is observed, which is a consequence of the initial focus on exploration of the state-action space previously mentioned. Then, over the course of 10^6^ learning steps, the mean switching time reduces to around 240 ps. The direct relationship between the switching time and the accumulated reward is readily demonstrated in Figure 7. As the switching time decreases, the accumulated reward increases, indicating that the agent has learned a better policy to select actions which can switch the memory cell faster. It should be pointed out that single runs were able to achieve an even better policy, which resulted in a minimum switching time of about 146 ps.

**Figure 8:**
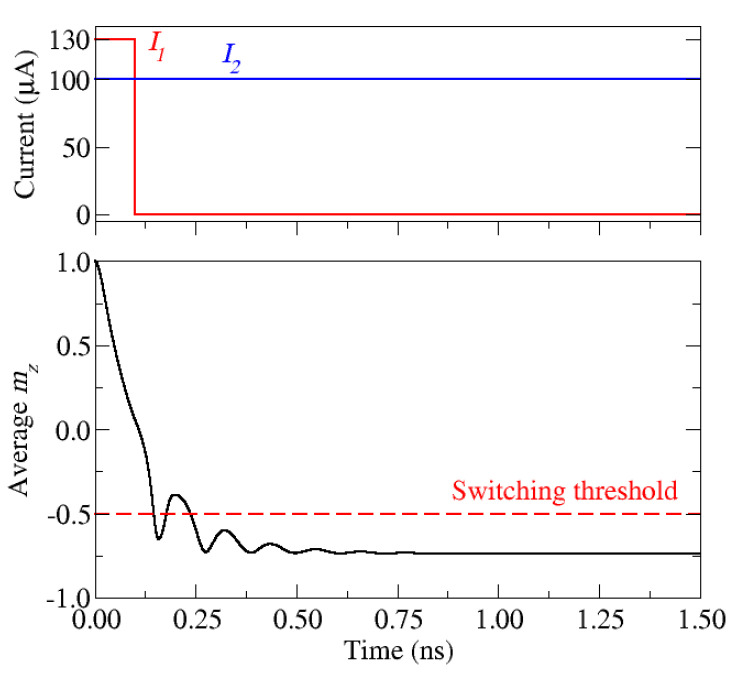
Pulse sequence learned by the DQN agent. *I*_1_ is the current amplitude of the first pulse applied to the NM1 wire and *I*_2_ is the current amplitude of the second pulse applied to the NM2 wire.

The pulse configuration learned by the DQN algorithm and the resulting magnetization dynamics are shown in Figure 8. The current pulses through the NM1 and the NM2 wire are turned on simultaneously right in the beginning of the simulation. After 100 ps, the first pulse is turned off and the magnetization component *m_z_* drops below the −0.5 threshold. Once this threshold is achieved, no further action is taken and the second current pulse is kept active for the rest of the simulation. This generates a SOT which acts on the FL under the NM2 wire, resulting in an average perpendicular magnetization component of about −0.8. Thus, the magnetization of the FL is not fully reversed to −1. This demonstrates the importance of the rewarding scheme and the general setup of the RL experiment. As the RL agent was rewarded for finishing the learning episode as fast as possible and the episode was considered finished as soon as the −0.5 threshold was reached, the agent learned how to achieve the threshold and did not take any action afterwards.

**Figure 9:**
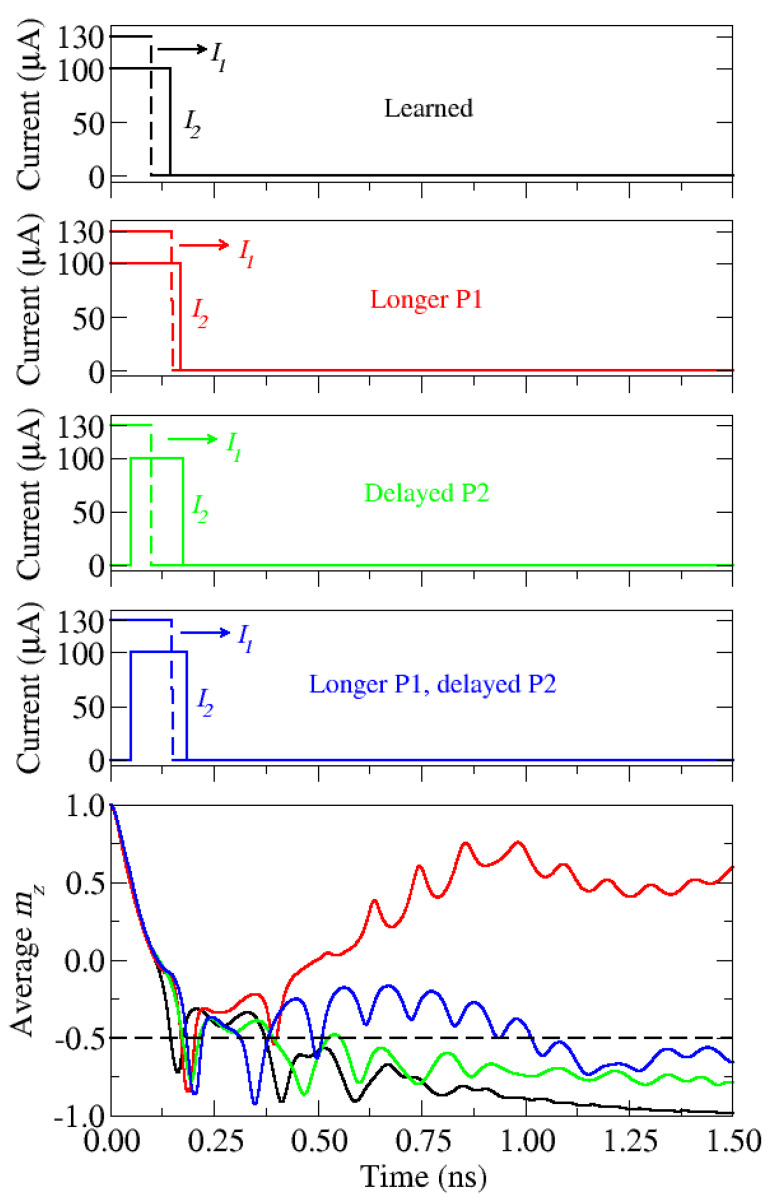
Comparison of different pulse configurations. The learned model is compared with modified ones. The learned pulse configuration leads to the fastest switching. *I*_1_ is the current amplitude of the first pulse, P1, applied to the NM1 wire and *I*_2_ is the current amplitude of the second pulse, P2, applied to the NM2 wire. The dashed line represents the switching threshold.

Figure 9 shows the dynamics of the magnetization component *m_z_* considering different variations of the learned pulse configuration. In the learned model, the second pulse is now switched off after *m_z_* = −0.5 is reached, which guarantees that the magnetization reversal is completed. The variations consisted of extending the first pulse and/or delaying the second pulse. A comparison of the magnetization dynamics with the learned model is given in Figure 9. One can observe that the learned configuration (black curve) leads indeed to the fastest switching. In turn, in the scenario with a longer first pulse, for which the pulses are almost perfectly overlapping, switching does not occur (red curve). The modified pulse sequences, represented by the green and blue curves, also lead to switching of the cell, however with longer switching times.

**Figure 10:**
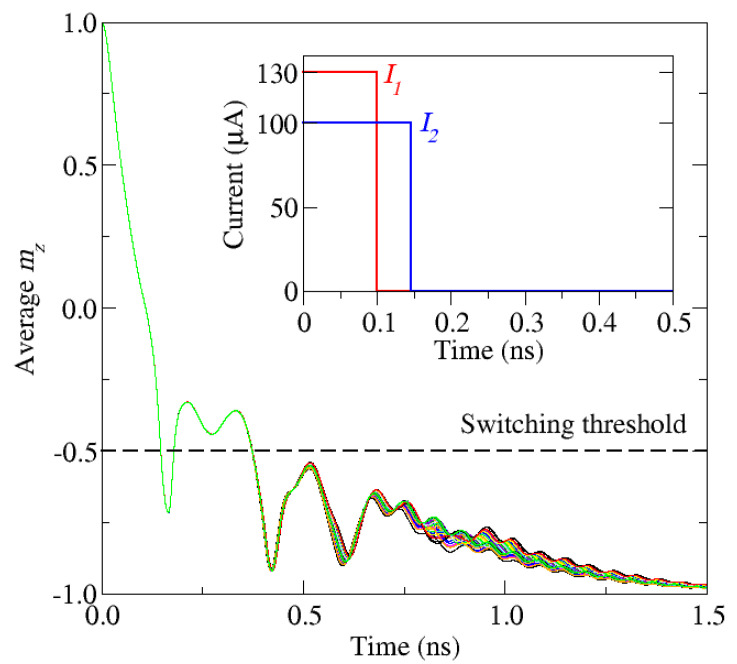
z-component of the magnetization of 50 switching realizations using the switching scheme found by the RL algorithm shown in the inset. *I*_1_ is the current amplitude of the first pulse and *I*_2_ is the current amplitude of the second pulse.

The robustness of the switching for the learned scheme is confirmed in Figure 10, for which 50 realizations under influence of the stochastic thermal field are reported. The variations between the different realizations are small and all of them switch, which shows that the learned scheme results in reliable and deterministic switching. It should be pointed out that, while the RL approach was able to find a scheme for which the switching time is 146 ps, the minimum switching time obtained from the previous manual configuration of the pulse was around 300 ps. This demonstrates the potential of the RL tool in combination with micromagnetic simulation for optimizing the two-pulse SOT switching scheme.

### 4.3. Impact of Parameter Variations

Although the fastest switching condition has been determined, variations of the pulse timing and/or of the process and material parameters of the magnetic FL can lead to slower or even non-deterministic switching. Thus, we now consider the impact of variations of the saturation magnetization and the anisotropy energy on the switching scheme.

**Figure 11:**
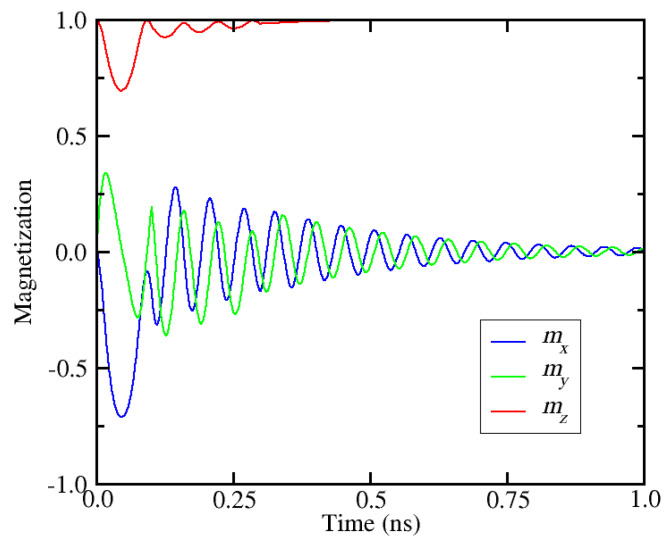
Magnetization dynamics for a cell with 5% variation of the perpendicular anisotropy energy and saturation magnetization in relation to the nominal values. The current density of the first pulse (2.7 × 10^12^ A/m^2^) is smaller than the critical current density, so the cell does not switch.

Figure 11 shows the x, y, and z components of the magnetization vector as a function of time for *K* = 8.8 × 10^5^ J/m^3^ and *M_S_* = 1.05 × 10^6^ A/m, which represent a variation of 5% in relation to the nominal parameter values. In this case the cell does not switch and, more importantly, one can observe that the perpendicular component of the magnetization (*m_z_*) does not reduce below 0.7. This means that the SOT generated by the applied current density of the first pulse (*j*_1_ = 2.7 × 10^12^ A/m^2^) is too weak to trigger the magnetization reversal. This can be explained by the fact that the variation of material parameters can change the critical current density for SOT switching. The above parameters lead to an increase of the critical current density, so that it becomes larger than the applied one. Thus, in order to switch this particular cell, the applied current has to be increased.

**Figure 12:**
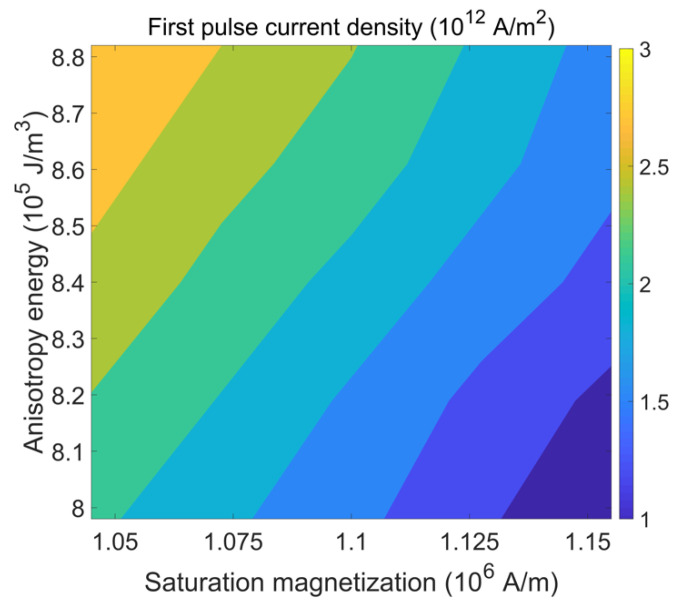
Minimum current density required for the first pulse to trigger reliable magnetization switching for cells with different combinations of saturation magnetization and anisotropy energy. The lowest current density is 1.0 × 10^12^ A/m^2^ (lower right corner) and the highest value is about 3.0 × 10^12^ A/m^2^ (upper left corner). The current density of the second pulse is 1.3 × 10^12^ A/m^2^ and both pulse durations are set to 200 ps.

Considering that different material parameter variations happen concurrently, one should expect that different cells of the same design undergoing the same fabrication process can require different current densities to trigger switching. Figure 12 shows the required current density for the first pulse to guarantee deterministic switching for various combinations of saturation magnetization and anisotropy energy. For 10% variation of the parameters, the minimum switching current density varies from 1.0 × 10^12^ A/m^2^ to about 3.0 × 10^12^ A/m^2^. These results indicate that, in order to switch all cells within the parameter spread, a current density of at least 3.0 × 10^12^ A/m^2^ has to be applied for the first pulse.

**Figure 13:**
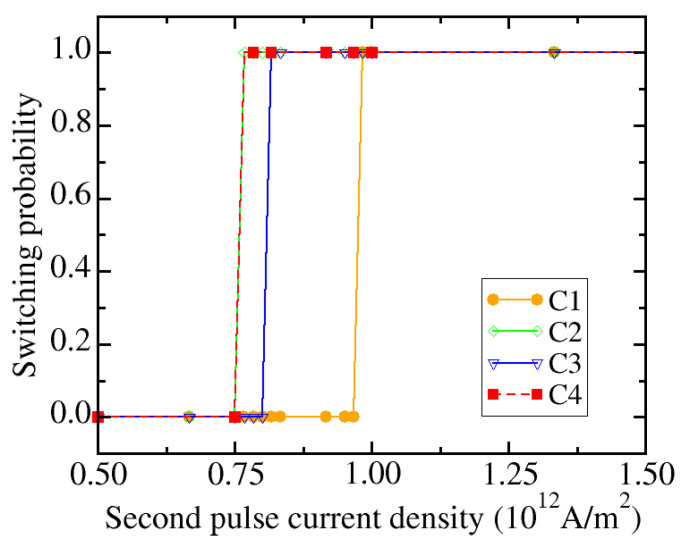
Switching probability as a function of the current density for the second current pulse, for four combinations of anisotropy energy and saturation magnetization. C1: *K* = 8.8 × 10^5^ J/m^3^, *M_S_* = 1.05 × 10^6^ A/m; C2: *K* = 8.8 × 10^5^ J/m^3^, *M_S_* = 1.16 × 10^6^ A/m; C3: *K* = 8.0 × 10^5^ J/m^3^, *M_S_* = 1.05 × 10^6^ A/m; C4: *K* = 8.0 × 10^5^ J/m^3^, *M_S_* = 1.16 × 10^6^ A/m. The current density of the first pulse is set to 3.0 × 10^12^ A/m^2^ and both pulse durations are 200 ps.

Next, the required current density for the second pulse is determined, as shown in Figure 13. We consider four combinations of anisotropy energy and saturation magnetization, denominated C1 to C4, which cover the variation space of Figure 12: *K* = 8.8 × 10^5^ J/m^3^, *M_S_* = 1.05 × 10^6^ A/m (C1), *K* = 8.8 × 10^5^ J/m^3^, *M_S_* = 1.16 × 10^6^ A/m (C2), *K* = 8.0 × 10^5^ J/m^3^, *M_S_* = 1.05 × 10^6^ A/m (C3), and *K* = 8.0 × 10^5^ J/m^3^, *M_S_* = 1.16 × 10^6^ A/m (C4), where C1 and C4 correspond to the two extreme cases, upper left and lower right corner, respectively, of Figure 12. In order to ensure 100% switching, the minimum current density required for the second pulse is about 1.0 × 10^12^ A/m^2^.

**Figure 14:**
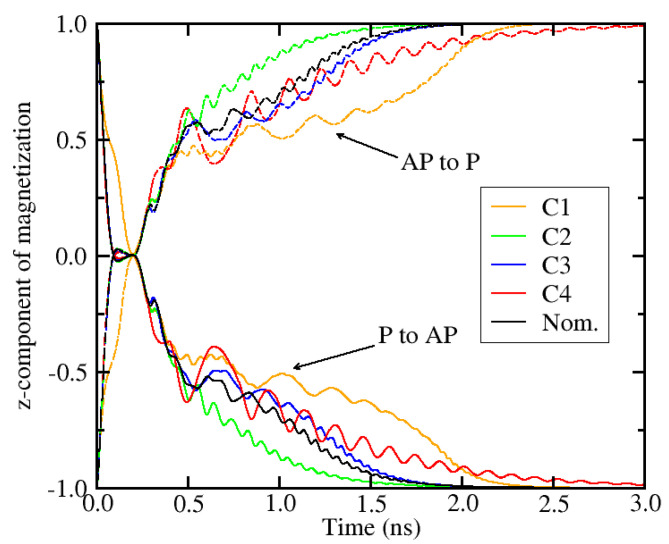
Average switching realizations from parallel to anti-parallel (P-AP) and anti-parallel to parallel (AP-P) for various combinations of anisotropy energy and saturation magnetization. C1: *K* = 8.8 × 10^5^ J/m^3^, *M_S_* = 1.05 × 10^6^ A/m; C2: *K* = 8.8 × 10^5^ J/m^3^, *M_S_* = 1.16 × 10^6^ A/m; C3: *K* = 8.0 × 10^5^ J/m^3^, *M_S_* = 1.05 × 10^6^ A/m; C4: *K* = 8.0 × 10^5^ J/m^3^, *M_S_* = 1.16 × 10^6^ A/m; Nom.: *K* = 8.4 × 10^5^ J/m^3^, *M_S_* = 1.1 × 10^6^ A/m. Each curve represents the average of 50 realizations, all of them resulting in successful switching.

The above analysis has allowed us to determine the minimum settings which guarantee 100% switching in the presence of cell-to-cell variations. Applying *j*_1_ = 3.0 × 10^12^ A/m^2^ and *j*_1_ = 1.3 × 10^12^ A/m^2^, the average switching realizations from parallel to anti-parallel (P-AP) as well as from anti-parallel to parallel (AP-P) configuration are reported in Figure 14, for the parameter combinations C1 to C4 and the nominal (Nom.) case, *K* = 8.4 × 10^5^ J/m^3^, *M_S_* = 1.1 × 10^6^ A/m. It should be pointed out that 50 realizations have been tested for each combination and all of them resulted in successful switching.

## 5. Conclusions

We developed a reinforcement learning approach in combination with micromagnetic simulations to optimize the switching of a spin-orbit torque memory cell. The magnetization switching is accomplished with a two-current pulse scheme and it is shown that, for sub-nanosecond operation, the switching probability strongly depends on the parameters of the applied current pulses. We demonstrated that the reinforcement learning setup can determine optimal sequence and timing parameters for the current pulses, which results in the fastest switching of the memory cell. This optimal pulse sequence yielded a switching time as short as 146 ps, remarkably shorter in comparison to a switching time of 300 ps for the manually configured pulse sequence. Based on our results, reinforcement learning is a promising tool to automate and further optimize spin-orbit torque switching based on the two-pulse scheme. We analyzed the impact of material parameter variations and showed that reliable switching can be guaranteed in the presence of cell-to-cell variations, provided that the current amplitude of the pulses is adjusted.

## Figures and Tables

**Table 1:** Simulation parameters. Heavy metal wires of b-tungsten and a magnetic FL of CoFeB on MgO are assumed [18].

Parameter	Value
Saturation magnetization, *M_S_*	1.1 × 10^6^ A/m
Exchange constant, *A*	1.0 × 10^−11^ J/m
Perpendicular anisotropy, *K*	8.4 × 10^5^ J/m^3^
Gilbert damping factor, *α*	0.035
Spin Hall angle, *θ_SH_*	0.3
Thermal stability factor, *Δ*	45
Free layer dimensions	40 nm × 20 nm × 1.2 nm
NM1: *w*_1_ × *l*	20 nm × 3 nm
NM2: *w*_2_ × *l*	20 nm × 3 nm
